# Oliceridine for Analgesia in Elderly Patients Undergoing Kyphoplasty: From a Clinical Trial

**DOI:** 10.1155/prm/2881787

**Published:** 2026-04-27

**Authors:** Chenfang Miao, Digui Weng, Chengzhao Liu, Qingqing Huang, Kongning Chen, Bin Zou

**Affiliations:** ^1^ Department of Anesthesiology, Mindong Hospital Affiliated to Fujian Medical University, Fuan, 355000, Fujian, China; ^2^ Department of Orthopaedics, Mindong Hospital Affiliated to Fujian Medical University, Fuan, 355000, Fujian, China

**Keywords:** age, analgesia, dose-response relationship, oliceridine, percutaneous kyphoplasty, sequential method

## Abstract

**Background:**

As a *G* protein‐biased μ‐opioid receptor agonist, oliceridine can offer a relatively effective and safe analgesia. However, its effect on patients suffering from kyphoplasty was unexplored.

**Aim:**

This study was aimed to determine the median effective dose (ED_50_) of oliceridine for analgesia during kyphoplasty in elderly patients across different age groups, providing clinical evidence for the optimal administration method.

**Methods:**

This prospective study included a total of 105 elderly patients with osteoporotic vertebral compression fractures (OVCFs) who underwent elective percutaneous kyphoplasty (PKP). The included subjects were divided into young age group (65–74 years old), middle age group (75–84 years old), and old age group (≥ 85 years old). The ED_50_ of oliceridine was determined by Dixon’s up–down sequential method. The manifestations of intraoperative analgesic insufficiency were defined as a Numerical Rating Scale (NRS) score ≥ 4, presence of body movement, an increased heart rate, or systolic and diastolic blood pressure > 20% from baseline during balloon dilation or bone cement injection.

**Results:**

When combined with remimazolam, the ED_50_ value of oliceridine for intraoperative analgesia during PKP was 0.035, 0.030, and 0.027 mg/kg in the young age group, middle age group, and old age group, respectively. The required ED_50_ in the young–old group was significantly higher than that in the old age group.

**Conclusion:**

The ED_50_ of oliceridine for intraoperative analgesia during PKP was varied from different age among these elderly patients. The lowest ED_50_ value in the old age group suggested that an increasing age might be associated with enhanced analgesic potency of oliceridine. The specific mechanism needs to be further elucidated in the future.

**Trial Registration:** Chinese Clinical Trial Register: ChiCTR2400095085

## 1. Introduction

With the acceleration of global aging, the incidence of osteoporotic vertebral compression fracture (OVCF) has shown a significant upward trend. As a first‐line surgical method for the treatment of OVCF, percutaneous kyphoplasty (PKP) has been widely performed in clinical practice [1]. However, the best anesthesia during the PKP has always been controversial. The operation is often accompanied by anxiety, high blood pressure, and body movements under a local anesthesia [2, 3], potentially causing an increased risk of surgical complications. Therefore, continuing to search for new analgesia drugs still has significant clinical significance.

Remimazolam is a new type of ultra‐short‐acting benzodiazepine, which exerts sedative effect by specifically activating *γ*‐aminobutyric acid type A (GABAA) receptor [4–6], which is recommended for intraoperative sedation in elderly patients with PKP [7]. However, patients with OVCF are often accompanied by thoracic kyphosis and pulmonary dysfunction [8]. There was significant difference in the sensitivity of opioids on those OVCF patients with various age groups [9], In recent years, some studies have shown that oliceridine, as a new *G* protein‐biased μ‐opioid receptor agonist, can selectively activate the *G* protein signaling pathway and inhibit the recruitment of *β*‐inhibitory proteins [10, 11], significantly reducing the risk of a series of opioid‐related adverse events (ORAEs), while providing effective analgesia during PKP procedure [12]. However, there is no adequate evidence on the application of oseltadine in PKP analgesia, and the impact of age on drug efficacy has not been ambiguous.

Therefore, the purpose of this study was to evaluate the effective dosage of oliceridine combined with remimazolam sedation in elderly patients with different age undergoing PKP surgery, possibly providing clinical evidence related to analgesia for elderly patients with OVCF.

## 2. Materials and Methods

### 2.1. Ethical Statement

This study was a prospective trial approved by the Ethics Committee of Mindong Hospital Affiliated to Fujian Medical University (ethics number: K2024110410).

### 2.2. Included Patients

**FIGURE 1:**
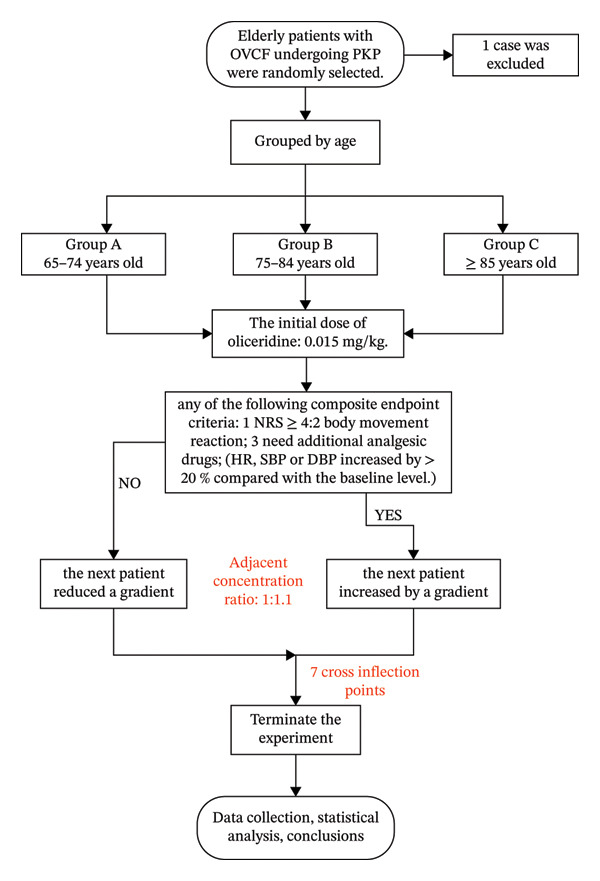
The flowchart of included subjects.

Elderly patients with OVCF who underwent elective PKP surgery from October 2024 to June 2025 were selected into our analysis. The inclusion criteria were defined as follows: (1) patients with age ≥ 65 years; (2) patients with body mass index (BMI) between 18 and 26 kg/m^2^; and (3) patients with an American Society of Anesthesiologists (ASA) physical status classification of II or III. Exclusion criteria were also defined as follows: (1) patients with painless or obsolete OVCF; (2) patients with preoperative coexistence of substantial lesions of important organs (such as cerebrovascular disease and cardiopulmonary disease); (3) patients with ASA grade ≥ IV or unable to tolerate surgery; and (4) patients with puncture site infection, known or suspected intestinal obstruction, inflammatory bowel disease, history of chronic pain, drug abuse or opioid dependence, history of allergy, abnormal coagulation function, participating in other clinical studies within 30 days, intraoperative general anesthesia, failed establishment of venous access, interference drugs used, or others. According to the age range, they were divided into the young elderly group (Group A, 65–74 years old), middle‐aged group (Group B, 75–84 years old), and elderly group (Group C, ≥ 85 years old) respectively, as shown in Figure 1.

### 2.3. Preanesthesia Preparation

Patients were instructed to fast for 8 h and refrain from clear fluids for 2 h prior before PKP surgery. Upon arrival in the operating room, peripheral intravenous access was established, and standard monitoring of vital signs was initiated. Oxygen was administered via nasal cannula at a flow rate of 2‐3 L/min. The attending anesthesiologist reviewed the anesthesia plan for each patient, and written informed consent for anesthesia was obtained to ensure full understanding and cooperation.

### 2.4. Anesthesia Protocol

Based on the existing literature evidence [10], an initial intravenous bolus of oliceridine (batch no: H20233509) at 0.015 mg/kg was administered slowly prior to surgery. Subsequent dose adjustment was made by using Dixon’s up‐and‐down sequential method (adjacent dose ratio of 1:1.1) according to the patient’s pain response [13]. The positive response was defined as the meeting any one of the following composite endpoints during balloon dilation or bone cement injection: (1) a Numerical Rating Scale (NRS) pain score ≥ 4; (2) observable body movement; (3) requirement for supplemental analgesics; or (4) an increase in heart rate (HR), systolic blood pressure (SBP), or diastolic blood pressure (DBP) of > 20% from baseline. Upon a positive response, the dose for the next patient was increased by one gradient. Otherwise, it was decreased. This sequence continued until seven nonpositive response crossover points were recorded. Similarly, remimazolam (batch no: 190905AK) was also injected intravenously at a loading dose of 0.2 mg/kg. All patients retained spontaneous breathing during the operation, and vital signs were continuously monitored. These patients were transferred to the postanesthesia care unit (PACU) and then returned to the ward after Steward score ≥ 4. No additional analgesic drugs were given after operation.

### 2.5. Observations

The main observations were the pain relief of patients during balloon dilatation or bone cement injection. Secondary outcomes included NRS pain score, SBP, DBP, HR, and SPO_2_ at different time points, dosage of vasoactive drugs (ephedrine and atropine), operation/anesthesia/recovery time, nausea and vomiting, respiratory depression (respiratory rate < 10 times/min or SPO_2_ < 90%), and circulatory fluctuation (blood pressure change > 20% or HR < 50 times/min).

### 2.6. Statistical Analysis

SPSS 27.0 and GraphPad Prism 9.5 were used for statistical analysis. The data with normal distribution were expressed as mean ± standard deviation, and variance analysis was used for comparison. The data with nonnormal distribution were expressed as median (Q1–Q3), and the Mann–Whitney *U* test was used for analysis. The categorical data were expressed as number (%), and the *χ* ^2^ test or Fisher method was used for the comparison. The median effective dose (ED_50_) and 95% confidence interval (CI) were calculated by Probit regression analysis. Chi square test from Probit analysis further helped us to determine the degree of goodness of fit between the model and data. The appropriate sample was evaluated as follows: *n* = [(Zα/2 × *σ*) ÷ *δ*]^2^. GraphPad Prism software was used to draw the sequence diagram and dose‐effect curve. *p* < 0.05 was considered to be statistically significant.

## 3. Results

### 3.1. Patient Characteristics

After one patient from the young elderly group was excluded due to general anesthesia during the operation, a total of 105 patients were included into our analysis (Group A, Group B, and Group C). As shown in Table 1, there were no significant differences in gender, BMI, and operation time among the three groups (*p* > 0.05). There were statistically significant differences in age and ASA classification (*p* < 0.05). Importantly, the number of negative reactions in Group A, Group B, and Group C was 12 cases, 14 cases, and 14 cases, respectively. The number of positive reactions was 23 cases, 21 cases, and 21 cases, respectively. The sequential test diagrams of each group are shown in Figure 2, respectively. This indicated that the rate of positive reactions in each group might be different.

**TABLE 1:** Characteristics of three patient groups.

Indicators	Male, *n* (%)	ASA physical status classification (II‐III)	BMI (kg/m^2^)	Age (years)	Operation time (min)
A Group	13 (37.1)	18–17	20.42 ± 0.87	67.3 ± 1.9	20.76 ± 1.37
B Group	15 (42.9)	9–26	19.97 ± 1.37	78.5 ± 2.2	20.17 ± 1.40
C Group	16 (45.7)	5–30	19.95 ± 1.07	86.6 ± 1.6	20.56 ± 2.84
*p*	0.760	0.003	0.144	< 0.001	0.463

*Note:* ASA: American Society of Anesthesiologists.

Abbreviation: BMI, body mass index.

FIGURE 2Dixon up‐and‐down plots.

**Figure:**
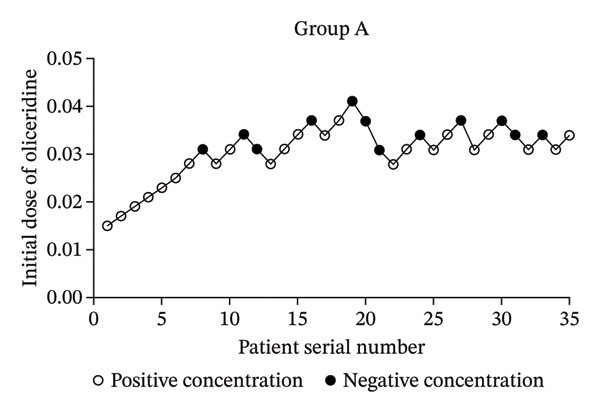
(a)

**Figure:**
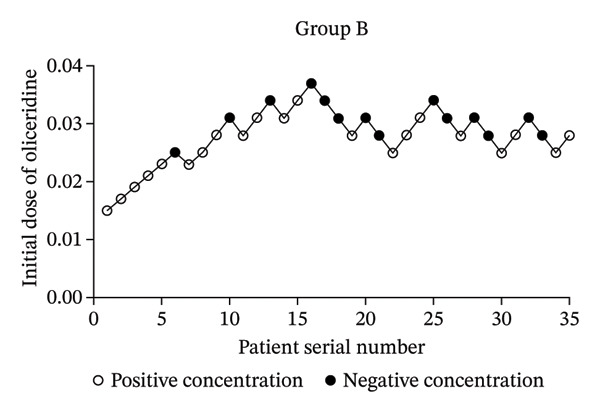
(b)

**Figure:**
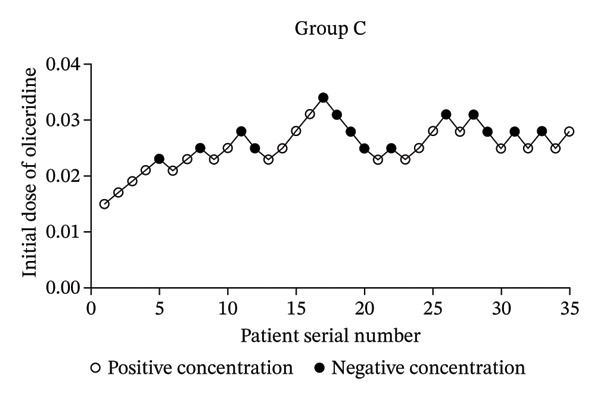
(c)

### 3.2. The Evaluation of ED_50_ Value for Inhibiting PKP Pain

**FIGURE 3:**
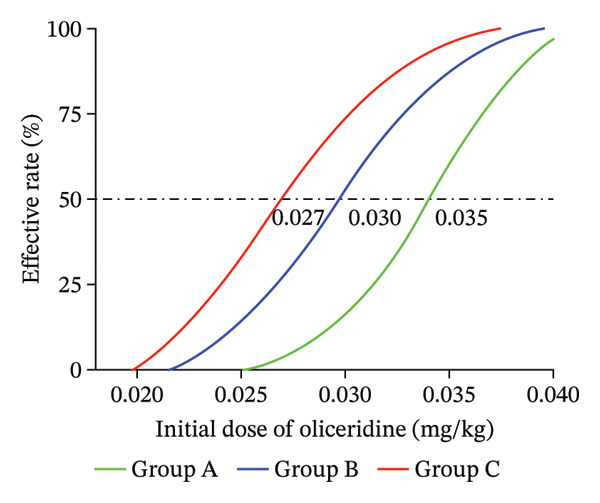
The potency intensity for oliceridine.

When combined with remimazolam, the ED_50_ value of oliceridine to inhibit PKP pain in Groups A, B, and C was 0.035 mg/kg (95% CI: 0.033–0.038), 0.030 mg/kg (95% CI: 0.029–0.032), and 0.027 mg/kg (95% CI: 0.026–0.029), respectively. The ED_50_ value of Group A was significantly higher than that of Group C (0.035 mg/kg vs. 0.027 mg/kg, *p* < 0.001). There was no significant difference between Group A and Group B (*p* = 0.238). There was no significant difference between Group B and Group C (*p* = 0.102). The dose‐effect relationship by fitting curve in each group is shown in Figure 3.

### 3.3. Observations

There was no significant difference in NRS pain score, HR, SBP, DBP, RR, and SPO_2_ among the three subgroups (*p* > 0.05). Compared with the baseline *T*_0_ time point, the pain NRS score, HR, SBP, and DBP of the three subgroups at *T*_1_–*T*_5_ time points were significantly different (all *p* < 0.05), as shown in Figure 4. No adverse reactions such as itching, headache, dizziness, nausea, and vomiting occurred in 105 patients. There was 1 case of hypotension in Group A and 1 case of hypotension and 1 case of respiratory depression in Group B. In Group C, there were 2 cases of hypotension and 1 case of respiratory depression. All adverse events were self‐limited and self‐relieved without special intervention. There was no significant difference in the incidence of adverse reactions, the use rate of vasoactive drugs, and patient satisfaction among the groups (all *p* > 0.05), as shown in Tables 2 and 3, respectively.

FIGURE 4Changes of NRS pain score, hemodynamic parameters, and oxygen saturation index at different time points in the three groups of patients (annotation: *T*_0_: before the start of anesthesia; *T*_1_: after bed posture; *T*_2_: trocar puncture vertebral body; *T*_3_: when bone cement is injected; *T*_4_: when pulling out the working casing; *T*_5_: immediately after operation. Compared with *T*_0_^∗^ *p* < 0.001).

**Figure:**
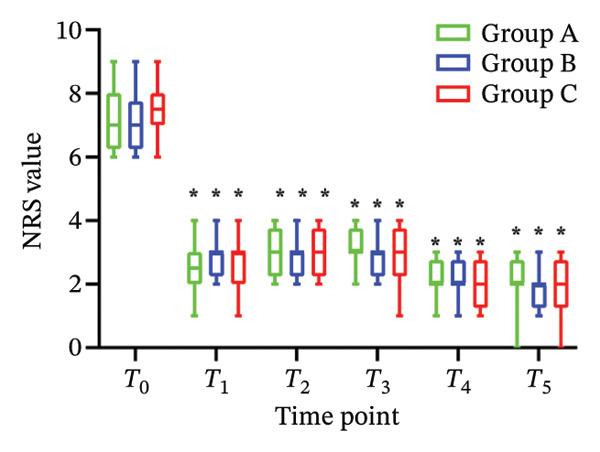
(a)

**Figure:**
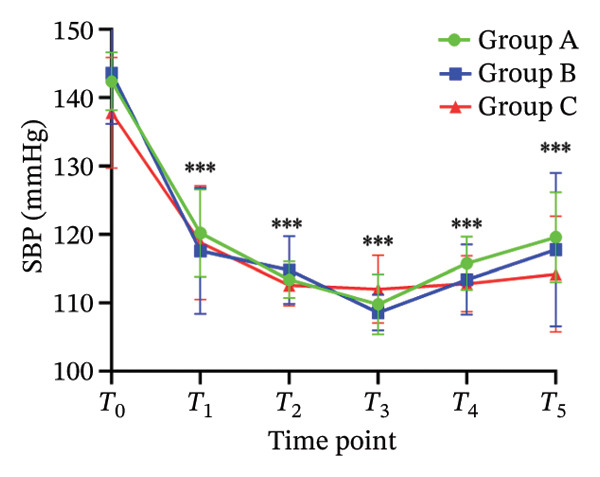
(b)

**Figure:**
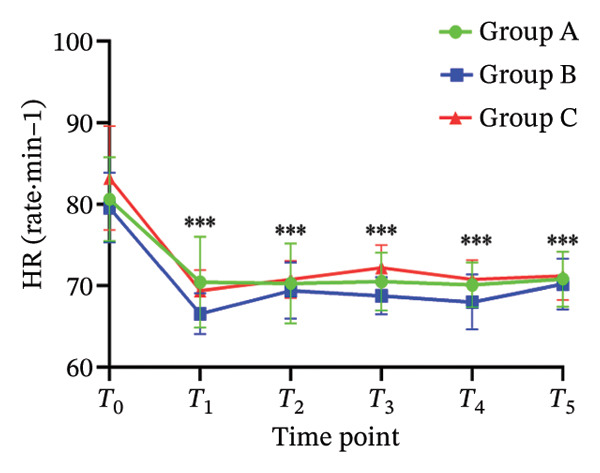
(c)

**Figure:**
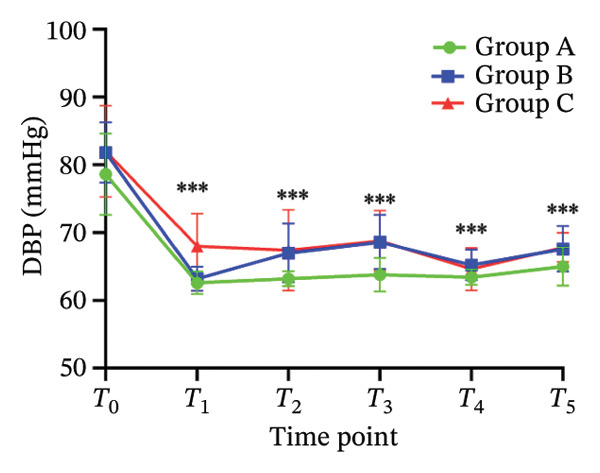
(d)

**Figure:**
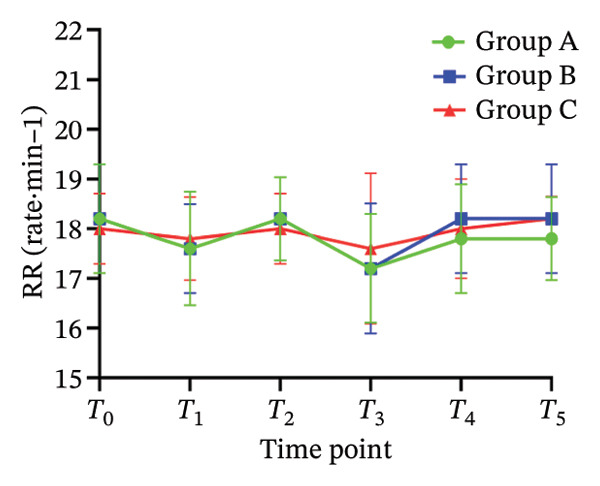
(e)

**Figure:**
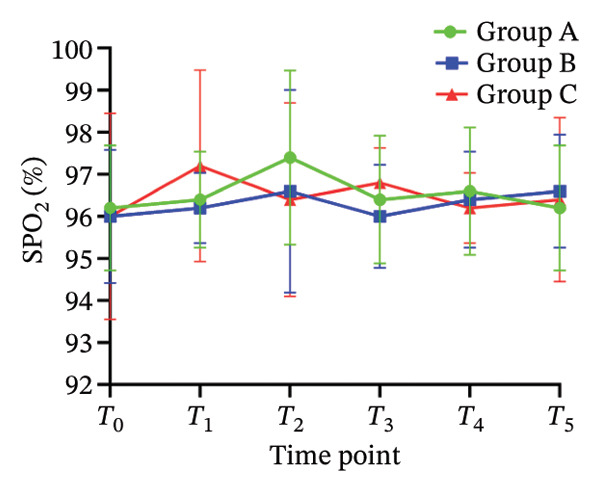
(f)

**TABLE 2:** Comparison of incidence of adverse events between the three subgroups.

Indicators	*n*	Itch, *n* (%)	Hypotension, *n* (%)	Respiratory depression *n* (%)	Headache, *n* (%)	PONV, *n* (%)	Use of ephedrine, *n* (%)	Use of atropine, *n* (%)
A Group	35	0	1 (5.7%)	0	0	0	0	1 (2.9%)
B Group	35	0	1 (5.7%)	1 (2.9%)	0	0	1 (2.9%)	0
C Group	35	0	2 (8.6%)	1 (2.9%)	0	0	1 (2.9%)	0
*p*	—	—	0.771	0.601	—	—	0.601	0.364

**TABLE 3:** Comparison of doctors and patients satisfaction between the three subgroups.

Indicators	Doctors’ satisfaction, *n* (%)					Patients’ satisfaction, *n* (%)				
	Very satisfied	Satisfied	Neutral	Not satisfied	Very dissatisfied	Very satisfied	Satisfied	Neutral	Not satisfied	Very dissatisfied
A Group	23 (65.7%)	10 (28.6%)	2 (5.7%)	0	0	26 (74.2%)	8 (22.9%)	1 (2.9%)	0	0
B Group	26 (74.2%)	8 (22.9%)	1 (2.9%)	0	0	27 (77.1%)	8 (22.9%)	0	0	0
C Group	27 (77.1%)	8 (22.9%)	0	0	0	28 (80%)	6 (17.1%)	1 (2.9%)	0	0
*p*	0.618					0.838				

## 4. Discussion

PKP has become the first‐line operation for the OVCF [1]. It was reported that the prevalence of OVCF in women over 80 years old can be as high as 36.6% [14]. Given the high risk of perioperative complications in elderly patients, the use of opioids should adhere to the principle of precision and individualization [15–18]. In this study, the calculated ED_50_ values of oliceridine for suppressing pain during PKP were 0.035 mg/kg in Group A, 0.030 mg/kg in Group B, and 0.027 mg/kg in Group C. These results indicated a statistically significant difference in the ED_50_ of oliceridine across age groups, with a marked decrease in dose requirement as patient age increased. This trend might indicate that oliceridine exhibits significant age‐dependent pharmacokinetic or pharmacodynamic variations in the elderly population.

Oliceridine, a *G* protein‐biased μ‐opioid receptor agonist, has demonstrated analgesic efficacy comparable to morphine with a more rapid onset in inhibiting moderate‐to‐severe pain under general anesthesia [11]. However, the effect of age on efficacy has not been specifically explored [19, 20]. Age is an important factor affecting the dose effect of various drugs [21]. Based on previous evidence on pharmacokinetic characteristics of elderly patients [10], the initial dose of oliceridine in each group was set to 0.015 mg/kg, and the pain relief during balloon dilatation and bone cement injection was selected as the main observation index. Elderly patients often exhibit reduced physiological functional reserve and a higher prevalence of comorbidities, which may lower the required analgesic dose threshold for effective pain control [21]. The results of this study demonstrated that the ED_50_ of oliceridine in Group C (≥ 85 years) was significantly lower than that in Group A (65–74 years) (0.027 vs. 0.035 mg/kg; *p* < 0.001), indicating a reduced dose requirement for oliceridine with advancing age. This phenomenon might be attributed to several age‐related physiological changes. For example, reductions in body water and muscle mass lead to a decreased volume of distribution for hydrophilic drugs, resulting in higher plasma concentrations with increasing age. Concurrently, a decline in plasma protein may increase the free fraction of the drug, thereby enhancing its pharmacological effect. Furthermore, degenerative change in the central nervous system can heighten sensitivity to anesthetic agents [18, 21], further reducing analgesic requirements. These findings were consistent with those reported by Kruijt et al., who also observed a significant influence of age on the effective dose of oliceridine combined with propofol in suppressing responses during gastroscopy [22]. Oliceridine produces analgesia through selective activation of the *G* protein signaling pathway within the endogenous opioid system. Studies have indicated that its potency for *G* protein activation is substantially higher than that of traditional opioids (EC_50_ = 8 nmol/L for oliceridine vs. 50 nmol/L for morphine) [19, 20]. Clinical evidence supported its efficacy in managing moderate‐to‐severe acute pain, as demonstrated in trials such as APOLLO‐1 (bunionectomy) and APOLLO‐2 (abdominal surgery) [23, 24]. Furthermore, research by Goudra et al. has confirmed its utility in sedation‐analgesia during gastrointestinal endoscopy [25]. Importantly, our results showed that the ED_50_ of oliceridine for effectively suppressing pain during PKP was significantly lower than the reported value for traditional opioids. Intraoperative pain NRS score, mean arterial pressure, and HR at all observed time points were significantly lower than baseline (*T*_0_) values (*p* < 0.001) across all groups. These results suggested that oliceridine can effectively alleviate pain induced by thermal and mechanical stimuli during balloon dilation and bone cement injection, thereby compensating for the limited nociceptive blockade achieved by local anesthesia alone in vertebral procedures.

Several limitations of this study should be noted. First, the absence of monitoring blood drug concentrations might have a certain impact on the accuracy of pharmacokinetic parameters, but this limitation does not weaken its reference value in clinical medication guidance. Second, this study is a single‐center and small‐sample clinical trial, and the results still need to be further verified by a larger sample size in multicenter studies. In spite of the above limitations, this study still provided valuable evidence for the exploration of ED_50_ of oliceridine among these patients suffering PKP. In addition, safety outcomes in this study were insufficient. For respiratory depression such as severity, management, monitoring parameters, and potential age‐related difference were missing. We will try our best to address the limitation in our subsequent research.

## 5. Conclusion

There was a significant difference in the ED_50_ value of combined remimazolam and oliceridine for PKP analgesia in elderly patients of different age groups. The ED_50_ of the elderly patients was significantly lower than that of the young elderly group, suggesting that age should be fully considered in clinical medication, and individualized medication strategy should be implemented to further improve the safety, while ensuring the analgesic effect during PKP surgery.

## Author Contributions

Chenfang Miao: data curation (equal); formal analysis (equal); investigation (equal); project administration (equal); and writing–original draft (lead). Digui Weng: data curation (equal) and project administration (equal). Chengzhao Liu: data curation (equal) and project administration (equal). Qingqing Huang: project administration (equal) and data curation (equal). Kongning Chen: data curation (equal). Bin Zou: data curation (equal); formal analysis (equal); investigation (equal); project administration (equal); and writing–review and editing (equal).

## Funding

This study was supported by Startup Fund for scientific research, Fujian Medical University (Grant number: 2024QH1258).

## Ethics Statement

Ethical approval was granted by the Ethics Committee of Mindong Hospital Affiliated to Fujian Medical University.

## Conflicts of Interest

The authors declare no conflicts of interest.

## Data Availability

All raw data and code are available from the corresponding author upon reasonable request.
